# Anemia and its associated factors among school-age children living in different climatic zones of Arba Minch Zuria District, Southern Ethiopia

**DOI:** 10.1186/s12878-019-0137-4

**Published:** 2019-04-23

**Authors:** Eshetu Zerihun Tariku, Getaneh Alemu Abebe, Zeleke Aschalew Melketsedik, Befikadu Tariku Gutema, Nega Degefa Megersa, Muluken Bekele Sorrie, Feleke Gebremeskel Weldehawariat, Eskeziyaw Agedew Getahun

**Affiliations:** 1 Department of Public Health, Arba Minch University, Arba Minch, Ethiopia; 2 Department of Medical Laboratory Science, Bahir Dar University, Bahir Dar, Ethiopia; 3 Department of Nursing, Arba Minch University, Arba Minch, Ethiopia

**Keywords:** Anemia, School-age children, Altitude, Ethiopia

## Abstract

**Background:**

Anemia, defined as a low blood hemoglobin concentration, has been shown to be a major public health concern in low-income countries like Ethiopia. School-age children are the most vulnerable population groups for anemia. The aim of this study was to assess the prevalence of anemia, with consideration of altitudinal variations, and to identify factors associated with anemia among school-age children.

**Methods:**

A community-based cross-sectional study was conducted from April to May 2017 among randomly selected 391 school-age children (6 to 14 years) in Arba Minch Health and Demographic Surveillance Site, Southern Ethiopia. Hemoglobin concentration was measured on the spot using portable hemoglobinometer (HemoCue Hb 201). The hemoglobin cut off values, adjusted for child age and altitude, were used to define anemia. Stool microscopic examination was done for investigation of intestinal parasites. A binary logistic regression model was used to assess the possible association of independent and outcome variables.

**Results:**

The overall prevalence of anemia was 37.3% (146); (95% CI: 32.5, 42.2). Among those who were anemic, 110 (28.1%) and 35 (9%) had mild (Hb 11–11.4 g/dl for children age from 6 to 11 years and 11–11.9 g/dl for children age from 12 to 14 years) and moderate (Hb 8–10.9 g/dl) anemia respectively. A single case of severe (Hb < 8 g/dl) anemia was identified. Fifty-seven (46.3%) of children living in an altitude ≥ 2500 m above sea level were anemic. Anemia was higher among children who were positive for intestinal parasitic infections (AOR = 3.30, 95% CI: 2.04, 5.35) and children not-enrolled to schools (AOR = 2.05, 95%CI: 1.26, 3.32). Anemia was less common among children who had no habit of eating vegetables in the last week prior to the survey (AOR = 0.35, 95%CI: 0.14, 0.84).

**Conclusions:**

More than one-third of school-age children were suffering from anemia. Intestinal parasitic infections and school non-enrollment were among the major factors associated with anemia among school-age children in the study area. Interventions, focusing on identified contributing factors need to be implemented by integrating with other school or community-based health programs.

## Background

Under-nutrition is one of the reasons behind high child mortality rates in developing countries. More than ten million children die each year, most from preventable causes and almost all in poor countries [1]. In Ethiopia, School-Age Children (SAC) are affected by a wide range of health and nutrition-related problems that constraint their ability to thrive and benefit from education [2]. Severe malnutrition is reported as the first leading cause of death among children aged 5 to 14 years old [3].

Anemia, defined as a low blood hemoglobin concentration, has been shown to be a public health problem that affects low-, middle- and high-income countries [4]. More than 40% SAC in developing countries are suffering from anemia and it is considered a severe public health problem. Sub-Saharan African countries shared a greater burden of the problem [5–7].

Based on pocket studies conducted in different parts of Ethiopia, the prevalence of anemia among SAC ranges from 23 to 38% [8–10]; while, the national level prevalence is not well documented.

Anemia has significant adverse health consequences, as well as adverse impacts on social and economic development [4]. It affects growth and energy levels adversely (5). It can also lead to reduced muscle function and work capacity; decreased school performance and increased school absenteeism in SAC [11, 12].

Anemia may result from a number of causes, with the most significant contributor being an iron deficiency (50%) [13]. Hookworms, which feed on their host’s blood and cause bleeding of the small intestine as well, are contributors of anemia. Recent studies have shown a marked increase in the prevalence of anemia caused by hookworm infection at age 5 to 14 years**,** particularly in tropical regions where hookworm prevalence is high [4, 5, 14]. Although iron-deficiency is the most common etiology of anemia globally, causes of anemia widely vary by geography, age group, and sex [14].

At elevations above 1000 m, hemoglobin concentration increases as an adaptive response to the lower partial pressure of oxygen and reduced oxygen saturation of blood. Therefore, the altitude of residence site of healthy individuals is critical for the definition of anemia. Hence, the altitude, along with other variables, should be taken into account while interpreting hemoglobin values in population-based surveys [15]. However, most of the previous studies were describing the burden of anemia without considering the relationship between altitude and hemoglobin concentration. Many studies were also institution based and might be subjected to unobserved confounding factors.

Moreover, for effective treatment and prevention of nutritional problems, local investigation of the problems has paramount importance, taking into account the prevalence and specific risk factors in a given setting and population group [15]. The aims of the present study were, therefore, to assess the prevalence of anemia, with consideration of altitudinal variations, and to identify factors associated with anemia among SAC.

## Methods

### Study design and setting

A community-based cross-sectional study was conducted from April to May 2017 among SAC (6 to 14 years old) in Arba Minch Health and Demographic Surveillance Site (AM-HDSS), Arba Minch Zuria district, Southern Ethiopia. Arba Minch Zuria district has a total of 31 kebeles, among which 9 kebeles are included as the site for AM-HDSS. These nine kebeles, which are assumed to be the representative of all 31 kebeles in the district, were selected by stratifying based on their climatic zone (all the three different climatic zones: high land, midland and lowland, are included) and, in addition, one semi-urban kebele was also included. The HDSS has been running by Arba Minch University, Demographic and Health Research Center. The altitude of nine kebeles ranges from 1128 to 2866 above sea level. According to the HDSS report of January 2016, a total of 74, 157 individuals live in the surveillance site, of whom 20,062 (27.1%) were aged between 6 and 14 years.

### Sample size and sampling technique

The sample size was estimated using single population proportion formula through assumptions of the prevalence of anemia among SAC in Jimma, which was 37.6% [16] at 95% confidence level (CI), 5% margin of error and adding 10% for non-responses. Finally, the study recruited a total of 397 children. All children aged 6 to 14 years old living in the nine kebeles of AM-HDSS were included as a source population. To select the study participants, AM-HDSS database, which contains the list of all individuals in the kebeles with their date of birth, individual and household identification, was used as a sampling frame. Then, considering the number of children living in each kebele, study participants were proportionally allocated. Study participants were selected following a simple random sampling technique using Stata version 14 software.

### Data collection methods

Data were collected using structured, pretested Amharic version questionnaires. The study instruments included two sets of structured questionnaires. One was administered for children, and another for their family (children guardian preferably the mother). Data on family wealth, as a measure of economic status, was collected by asking ownership of selected assets that are common in the local area based on Ethiopian Demographic and Health Survey (EDHS) 2016 wealth index variables [17]. The household food insecurity level was measured using Household Food Insecurity Access Scale (HFIAS), which is a structured, standardized and validated tool that developed by Food and Nutrition Technical Assistance Project (FANTA) [18]. The child dietary intake pattern, for assessing the child dietary diversity, was measured by a qualitative recall of all foods consumed by each child during the previous 24 h based on Food and Agriculture Organization (FAO) Guideline. In addition, to assess the usual nutrient intake of the child, the frequency of consumption of certain nutrients 1 week prior to the survey was recorded [19]. Altitude, where each child resides, was measured to describe the malaria transmission pattern in the study area and to aid in the adjustment of the hemoglobin concentration of the child.

Weights of children were measured to the nearest 0.1 kg using calibrated portable electronic digital scale (Seca, Germany model) and heights were measured to the nearest 0.1 cm using a portable height-measuring board with a sliding head bar following standard anthropometric techniques [20].

#### Hemoglobin measurement

Trained medical laboratory technicians collect capillary blood samples in order to measure hemoglobin concentration of each child on the spot using portable hemoglobinometer (HemoCue Hb 201) following standard protocols as explained elsewhere [8].

#### Stool examination

Stool examination for microscopic detection of intestinal parasites was also conducted following standard protocol as explained elsewhere [21]. Detail of stool examination and findings are available in our previous publication [22].

### Data quality control

A questionnaire was prepared in English and translated into Amharic language and again translated back to English. It was pretested by recruiting 20 children and their guardians in one of HDSS kebeles on households which were not selected for actual data collection. Data collectors were given 2 days of intensive training on the method of identifying the household, anthropometric measurements, blood and stool sample collection and examination, ethical issues and the purpose of the study. Hemoglobin measurement and stool microscopic examination were done following standard protocols. Investigators were supervising all aspects of data collection throughout the data collection period.

### Data analysis

Data were entered using Epi Data version 3.1. For further analysis, the data was exported to Statistical Package for Social Science (SPSS) version 22 software. Descriptive analysis was indicated using numerical summary measures. Households who experienced none of the food insecurity (access) conditions or just experiences worry, but rarely in the past 4 weeks were labeled as ‘food secured, while the remaining were labeled as food insecure [18]. To calculate the child dietary diversity score, certain food groups were aggregated and the mean score was used to classify child food intake as adequate or not [19].

The blood hemoglobin cutoff values were adjusted for altitude before determining the children either as anemic or not. The adjustment was made based on the application of formula: Hb adjustment = − 0.32 × (altitude in meters × .0033) + 0.22 × (altitude in meters × .0033)^2^. Then, the adjustment was subtracted from the measured hemoglobin concentration at the relevant altitude to get the sea-level value [23–25]. The hemoglobin concentration cutoff points for anemia were 11.5g/dl for children age from 6 to 11 years and 12 g/dl for children age from 12 to 14 years. Those children with hemoglobin concentration below 8 g/dl and between 8 and 10.9g/dl were further classified as severe and moderate anemia, respectively. The remaining anemic children with hemoglobin concentration between 11 and 11.4g/dl (for children age from 6 to 11 years) and 11–11.9 g/dl (for children age from 12 to 14 years) were categorized as mild anemia [26].

The wealth index was constructed via Principal Component Analysis (PCA) method [27]. For anthropometric data analysis, standard deviation (Z-scores) scores were obtained by the World Health Organization (WHO) Anthro Plus software. Children whose Height for Age z-score (HAZ) and BMI for Age z-score (BAZ) above-2SD scores were considered as well-nourished and those below -2SD scores as being malnourished (stunted and thin respectively) [28, 29]. Malaria transmission pattern in the study area was described based on the difference in altitude: malaria-free highland areas (above 2500 m altitude); highland fringe areas between 1500 and 2500 m affected by frequent epidemics; and lowland areas below 1500 m with seasonal patterns of transmission [30].

All covariates that were significant at *p*-value < 0.25 in bivariate analysis were considered for multivariable analysis to control possible confounders. To measure the strength of association between dependent and independent variables, Adjusted Odds Ratio (AOR) with 95% Confidence interval (CI) was calculated. Finally, the level of statistical significance was declared at *p*-value < 0.05. The fitness of the model was tested by Hosmer- Lemeshow goodness of fit test and the test revealed a *p*-value of 0.820 showing the model was fit.

## Results

### Socio-demographic and economic characteristics of study participants

A total of 391 SAC with their mothers/caregivers were participated in making a response rate of 98.5%. More than half of respondents, (65.0%) were in the age group of 6–11 years. The mean ± SD of the age of children was 10.1 ± 2.6 years while that of parents/ caregivers was 37 ± 8.7 years. Nearly half (50.1%) of the participating children were male and 223 (57.0%) were enrolled in school. Concerning the educational status of parents, 257 (65.7%) of mothers/ caregivers, had no formal education while 132 (33.8%) of fathers had formal education. A total of 370 (94.6%) mothers were housewives while 133 (34%) of the fathers were farmers.

In the study area, 130 (33.25%) of households were in the poor wealth terciles; 206 (52.7%) of households were food insecure and 159 (40.7%) of surveyed households were residing in kebeles with an altitude < 1500 m where seasonal transmission of malaria is common (Table 1).

**Table 1:** Distribution of socio-demographic and economic characteristics of school age children with and without anemia (*n* = 391), Arba Minch Zuria District, Southern Ethiopia, April to May 2017

Variables	Categories	Anemic		COR (95%CI)	*p*-value
		Yes (%)	No (%)		
Age of child (in years)	6–11	100 (39.4)	154 (60.6)	1.26 (0.83–1.98)	0.259
	12–14	46 (33.6)	91 (66.4)	1	
Child sex	Male	72 (36.7)	124 (63.3)	1	
	Female	74 (37.9)	121 (62.1)	1.05 (0.70–1.59)	0.804
School Enrolment	Enrolled	69 (30.9)	154 (69.1)	1	
	Non enrolled	77 (45.8)	91 (54.2)	1.89 (1.25–2.86)	0.003*
Age of parent/ care giver	18–35	68 (35.4)	124 (64.6)	1	
	36–45	58 (39.7)	88 (60.3)	1.20 (0.77–1.87)	0.417
	> 45	20 (37.7)	33 (62.8)	1.11 (0.59–2.07)	0.755
Sex of parent/ care giver	Male	54 (45.8)	64 (54.2)	1	
	Female	92 (33.7)	181 (66.3)	0.60 (0.39–0.94)	0.024*
Educational status of mother	No formal education/unable to read and write	114 (38.6)	181 (61.4)	1.26 (0.78–2.05)	0.351
	Formal education	32 (33.3)	64 (66.7)	1	
Educational status of father	No formal education	103 (39.8)	156 (60.2)	1.37 (0.88–2.12)	0.165*
	Formal education	43 (32.6)	89 (67.4)	1	
Occupation of mother/ care giver	Housewife	141 (38.1)	229 (61.9)	1.97 (0.71–5.50)	0.195*
	Working	5 (23.8)	16 (76.2)	1	
Occupation of father	Government employee	7 (58.3)	5 (41.7)	2.42 (0.75–7.76)	0.138*
	Private employee	139 (36.7)	240 (63.3)	1	
Family size (in number)	< 4	5 (27.8)	13 (72.2)	1	
	> = 4	141 (37.8)	232 (62.2)	1.58 (0.55–4.53)	0.394
Family wealth terciles	Poor	53 (40.8)	77 (59.2)	1.39 (0.84–2.31)	0.199*
	Medium	50 (38.2)	81 (61.8)	1.25 (0.75–2.08)	0.391
	Rich	43 (33.1)	87 (66.9)	1	
Household food security status	Food secured	68 (36.8)	117 (63.2)	1	
	Food insecured	78 (37.9)	128 (62.1)	1.05 (0.70–1.58)	0.821
Altitude in meter (based on malaria transmission)	< 1500	49 (30.8)	110 (69.2)	0.52 (0.32–0.84)	0.008*
	1500-2500	40 (36.7)	69 (63.3)	0.67 (0.40–1.14)	0.138*
	Malaria free (≥ 2500)	57 (46.3)	66 (53.7)	1	

*COR* Crude Odds Ratio, *CI* Confidence Interval; * = *p*-value < 0.25

### Anthropometric status, dietary and clinical characteristics of study participants

Out of all children included in this study, 163 (41.9%) were with height for age < −2Z score and 31 (8.0%) were with BMI for age < −2Z score. The detail on the anthropometric status of the child is found in our previously published article [31]. The stool samples were collected from 391 children among which 182 (46.5%) of children were positive for intestinal parasites and the remaining 209 (53.5) were negative. *Ascaris lumbricoides* (14.2%) and hookworms (14.1%) being the predominant species. Only 16 (4.1%) of children reported the presence of illness 2 weeks prior to the survey. Nearly all children, 364 (93.1%) had no habit of consumption of meat or meat products 1 week prior to the survey. More than half of children, 238 (60.9%), had a Dietary Diversity Score (DDS) of ≥ 4 food groups (Table 2).

**Table 2:** Distribution of child health, dieting habit and environmental characteristics of school age children with and without anemia (*n* = 391), Arba Minch Zuria District, Southern Ethiopia, April to May 2017

Variables	Category	Anemic		COR (95% CI)	*p*-value
		Yes (%)	No (%)		
Presence of illness in the last two weeks prior to the survey	Yes	2 (12.5)	14 (87.5)	0.23 (0.05–1.02)	0.054*
	No	144 (38.4)	231 (61.6)	1	
Current IP infection	Positive	87 (47.8)	95 (52.2)	2.33 (1.53–3.54)	0.000*
	Negative	59 (28.2)	150 (71.8)	1	
HAZ < -2	Yes	69 (42.3)	94 (57.7)	1.42 (0.94–2.15)	0.097*
	No	77 (34.1)	149 (65.9)	1	
BAZ < -2	Yes	13 (41.9)	18 (58.1)	1.22 (0.58–2.57)	0.598
	No	133 (37.2)	225 (62.8)	1	
Number of days the child eat fruit in the last week prior to the survey	Do not eat fruits	94 (44.1)	119 (55.9)	1.81 (1.01–3.22)	0.046*
	<=3 days	31 (28.4)	78 (71.6)	0.91 (0.47–1.76)	0.776
	> 3 days	21 (30.4)	48 (69.6)	1	
Number of serving of fruit the child eat on one of those days	< 3 servings	44 (28.0)	113 (72.0)	0.63 (0.25–1.63)	0.344
	> = 3 servings	8 (38.1)	13 (61.9)	1	
Number of days the child eat vegetables in the last week prior to the survey	Do not eat vegetables	13 (24.5)	40 (75.5)	0.44 (0.22–0.86)	0.017*
	<=3 days	41 (33.3)	82 (66.7)	0.67 (0.42–1.06)	0.088*
	> 3 days	92 (42.8)	123 (57.2)	1	
Number of serving of vegetables the child eat on one of those days	< 3 servings	127 (39.8)	192 (60.2)	1.43 (0.53–3.87)	0.477
	> = 3 servings	6 (31.6)	13 (68.4)	1	
Number of days the child eat meat/meat products in the last week prior to the survey	Do not eat meat/MPs	136 (37.4)	228 (62.6)	0.60 (0.04–9.61)	0.716
	<=3 days	9 (36.0)	16 (64.0)	0.56 (0.03–10.12)	0.696
	> 3 days	1 (50.0)	1 (50.0)	1	
Child DDS	< 4 food groups	65 (42.5)	88 (57.5)	1.43 (0.94–2.17)	0.092*
	> = 4 food groups	81 (34.0)	157 (66.0)	1	
Latrine availability at home	Yes	138 (36.9)	236 (63.1)	1	
	No	8 (47.1)	9 (52.9)	1.52 (0.57–4.03)	0.400
Source of drinking water	Safe (pipe, protected well or spring)	127 (37.5)	212 (62.5)	1	
	Unsafe (river, unprotected well or spring)	19 (36.5)	33 (63.5)	0.96 (0.52–1.76)	0.898
Waste disposal system	Appropriate (burning, pit, garbage can)	111 (35.5)	202 (64.5)	1	
	Inappropriate (open field/no disposal system)	35 (44.9)	43 (55.1)	1.48 (0.90–2.45)	0.126*
Ever sleeps under bed net	Yes	52 (34.0)	101 (66.0)	1	
	No	94 (39.5)	144 (60.5)	1.27 (0.83–1.94)	0.272
Ever received health or nutrition related information	Yes	61 (33.3)	122 (66.7)	1	
	No	85 (40.9)	123 (59.1)	1.38 (0.91–2.09)	0.125*

*COR* Crude Odds Ratio, *CI* Confidence Interval; * = *p*-value < 0.25

### Prevalence of anemia among study participants

Before the adjustment of children hemoglobin concentration for altitude, the mean ± SD hemoglobin level of study participant was 13.1 ± 1.8. After adjustment, the mean ± SD hemoglobin level of study participant was 12.3 ± 1.8. The overall prevalence of anemia among SAC was 37.3% (146); (95% CI: 32.5, 42.2). Among those who were anemic, 110 (28.1%) (95% CI: 23.8, 33.0) had mild form of anemia, 35 (9%) (95% CI: 6.1, 11.8) moderate form of anemia, and a single case of severe anemia (0.3%) (95% CI: 0.0, 0.8) was identified. The prevalence of anemia was 57 (46.3%), 40 (36.7%) and 49 (30.8%) in an altitude ranges of ≥2500 m, between 1500 and 2500 m and <  1500 m respectively, even though, the difference is not statistically significant when adjusted for other covariates.

### Factors associated with anemia among study participants

The present study identified major modifiable associated factors for anemia. Children who were positive for intestinal parasitic infections were 3.3 times more likely to be anemic than those negative for intestinal parasitic infection (AOR = 3.30, 95% CI: 2.04, 5.35). The likelihood of anemia was also higher among children not-enrolled in schools (AOR = 2.05, 95%CI: 1.26, 3.32) than those children who were enrolled. When compared with children who had the habit of eating vegetables for more than 3 days in 1 week prior to the survey, anemia was less common among children who had no habit of eating vegetables in 1 week prior to the survey (AOR = 0.35, 95%CI: 0.14, 0.84) (Table 3).

**Table 3:** Multivariable logistic regression analysis showing factors associated with anemia among school aged children (*n* = 391), Arba Minch Zuria District, Southern Ethiopia, April to May 2017

Variables	Categories	Anemic		AOR (95%CI)	*p*-value
		Yes (%)	No (%)		
School Enrolment	Enrolled	69 (30.9)	154 (69.1)	1	
	Non enrolled	77 (45.8)	91 (54.2)	2.05 (1.26–3.32)	0.004*
Sex of parent/ care giver	Male	54 (45.8)	64 (54.2)	1	
	Female	92 (33.7)	181 (66.3)	0.65 (0.36–1.14)	0.133
Educational status of father	No formal education	103 (39.8)	156 (60.2)	0.87 (0.47–1.63)	0.666
	Formal education	43 (32.6)	89 (67.4)	1	
Occupation of mother/ care giver	Housewife	141 (38.1)	229 (61.9)	2.09 (0.64–6.76)	0.220
	Working	5 (23.8)	16 (76.2)	1	
Occupation of father	Government employee	7 (58.3)	5 (41.7)	3.41 (0.84–13.96)	0.088
	Private employee	139 (36.7)	240 (63.3)	1	
Family wealth terciles	Poor	53 (40.8)	77 (59.2)	0.63 (0.23–1.73)	0.369
	Medium	50 (38.2)	81 (61.8)	0.70 (0.29–1.69)	0.422
	Rich	43 (33.1)	87 (66.9)	1	
Altitude in Meter (based on malaria transmission)	< 1500	49 (30.8)	110 (69.2)	0.66 (0.17–2.56)	0.546
	1500-2500	40 (36.7)	69 (63.3)	0.77 (0.33–1.81)	0.552
	Malaria free (> 2500)	57 (46.3)	66 (53.7)	1	
Presence of illness in the last two weeks prior to the survey	Yes	2 (12.5)	14 (87.5)	0.30 (0.06–1.57)	0.153
	No	144 (38.4)	231 (61.6)	1	
Current IP infection	Positive	87 (47.8)	95 (52.2)	3.30 (2.04–5.35)	0.000*
	Negative	59 (28.2)	150 (71.8)		
HAZ < -2Z score	Yes	69 (42.3)	94 (57.7)	1.22 (0.74–2.03)	0.443
	No	77 (34.1)	149 (65.9)		
Number of days the child eat fruit in the last week prior to the survey	Do not eat fruits	94 (44.1)	119 (55.9)	1.70 (0.57–5.06)	0.339
	<=3 days	31 (28.4)	78 (71.6)	1.08 (0.51–2.31)	0.842
	> 3 days	21 (30.4)	48 (69.6)	1	
Number of days the child eat vegetables in the last week prior to the survey	Do not eat vegetables	13 (24.5)	40 (75.5)	0.35 (0.14–0.84)	0.019*
	<=3 days	41 (33.3)	82 (66.7)	0.83 (0.43–1.63)	0.597
	> 3 days	92 (42.8)	123 (57.2)	1	
Child DDS	< 4 food groups	65 (42.5)	88 (57.5)	1.27 (0.66–2.44)	0.473
	> = 4 food groups	81 (34.0)	157 (66.0)	1	
Waste disposal system	Appropriate (burning, pit, garbage can)	111 (35.5)	202 (64.5)	1	
	Inappropriate (open field/no disposal system)	35 (44.9)	43 (55.1)	0.56 (0.22–1.42)	0.222
Ever received health or nutrition related information	Yes	61 (33.3)	122 (66.7)	1	
	No	85 (40.9)	123 (59.1)	1.19 (0.67–2.12)	0.546

*AOR* Adjusted Odds Ratio, *CI* Confidence Interval; * = *p*-value < 0.05

## Discussion

The present study revealed that about 37.3% of SAC were anemic. This finding is in agreement with the prevalence of anemia reported by a study from Jimma, Ethiopia (37.6%) [16]. It also concurs with a report from India (37.5%); even though the method used to diagnose anemia was based on clinical examinations [32].

The prevalence of anemia among SAC in the present study is higher than research finding from Somali and Kersa Ethiopia where the prevalence of anemia was 23.7 and 27.1% respectively [8, 10]. The higher prevalence of anemia in our study might be attributed to the difference in the study period, the hemoglobin cut-off value used to diagnose anemia (we considered hemoglobin adjustment for altitude), and a relatively higher proportion of children affected by intestinal parasites; which is one of the contributing factors for the development of anemia. In this study, the overall prevalence of anemia, without the adjustment, was 22.5%. This is an indication that the prevalence of anemia is underestimated if the hemoglobin concentration cut-off values are not adjusted for important variables like altitude. As expected, the prevalence of anemia reported by the present study is much higher than the prevalence reported elsewhere in developed countries like the United States, Colombia, and Mexico; which was below 12.0% [32]. The variation could probably attribute to the difference in socio-economic and demographic characteristics of study participants.

Infection and malnutrition have a bidirectional relationship. Chronic worm infections often make children malnourished, anemic and vulnerable to illnesses. The present study identified a statistically significant association between intestinal parasitic infection and child anemia. The odds of developing anemia were 3.3 times more common among children who were positive for intestinal parasitic infections than those who were tested negative. This is in agreement with evidence from the study conducted in Somali, Ethiopia [8]. This might be due to the fact that hookworms, which feed on their host’s blood and cause bleeding of the small intestine as well, are among the predominant parasites detected.

Children who had no habit of eating vegetables in a week prior to the survey were 65% less likely to be anemic compared to children who had the habit of eating vegetables for more than 3 days in a week. This could be explained by vegetables are among the non-heam dietary iron sources which have poor bioavailability, and their absorption can be influenced by different factors. Thus, unless the consumption of such dietary sources are supplemented with optimal nutrition education interventions, addressing the number of servings per day and about the type of foods that will potentially inhibit its absorption, the habit of consumption of vegetables alone cannot be a guarantee for reducing the risk of anemia [33, 34]. However, since the majority of resource-poor countries are using non-haem iron as the main source of dietary iron, the consumption of vegetables should be encouraged as it is among the good dietary groups for the population predominantly based on non-heme iron [35].

In this study, anemia was 2 times more common among children not-enrolled in schools than those children who were enrolled. This could be explained by the probability that children who were not enrolled in schools, in most rural areas, are widely engaged in activities that let them stay outside their home. Consequently, they may not have access to sufficient care and support from their families which could contribute to the depletion of their nutritional status [36].

Although the prevalence of anemia was higher (46.3%) among SAC residing in higher altitudes, the difference was not statistically significant (*p*-value: 0.552). Similarly, the present study did not find a statistically significant association between the child anthropometric status and the presence of anemia. The relatively low prevalence of thinness (8.0%) could be one reason for the absence of a statistically significant association. However, the multivariable logistic regression analysis showed that anemia was more common (42.3%) among stunted children (HAZ < -2) than their counterparts, non-stunted children (34.1%).

One of the strengths of this study is that it was community-based and can provide information about both enrolled and not-enrolled SAC. The study also tried to address multifactorial causes of anemia including investigation of the presence of intestinal parasitic infections. The application of standard criteria’s (like hemoglobin adjustment for altitude difference) was also the strength of the present study. Recall bias is one of the limitations of this study since some of the questions (food security questions) asked respondents to remember the event during the last 4 weeks. This was minimized by probing the respondents about the event. The other limitation of this study is it couldn’t tell us about a specific type of anemia since the detailed morphological investigation of red blood cells was not made.

## Conclusions

More than one-third of SAC was suffering from anemia. Intestinal parasitic infections and being not-enrolled in schools were found to be the major factors contributed to the development of anemia in the study area. The habit of consumption of vegetables within a week prior to the survey was also associated with anemia among SAC. Thus, interventions, focusing on identified contributing factors; need to be implemented by integrating with other school health programs to improve the nutritional status of SAC. Measures targeted at reducing the burden of intestinal parasitic infection should be strengthened to reduce the burden of anemia. Emphasis should also be given to improving children enrolment to schools. Nutrition education programs, mainly focusing on the consumption of dietary sources of iron, are beneficial to reduce the burden of anemia. In addition, further study to assess specific causes of anemia using detail morphological investigations is recommended.

## Abbreviations

AM-HDSS: Arba Minch Health and Demographic Surveillance Site
SAC: School Age Children
